# Impact of the COVID-19 pandemic in patients with a previous history of premature myocardial infarction

**DOI:** 10.1016/j.ajpc.2020.100128

**Published:** 2020-11-18

**Authors:** Meral Kayikcioglu, Ozlem Kuman Tuncel, Lale Tokgozoglu

**Affiliations:** a Ege University Medical Faculty, Department of Cardiology, İzmir, Turkey; b Ege University Medical Faculty, Department of Psychiatry, İzmir, Turkey; c Hacettepe University Medical Faculty, Department of Cardiology, Ankara, Turkey

**Keywords:** *Anxiety*, *COVID-19*, *Premature atherosclerosis*, *Coronary artery disease*, *Lifestyle changes*, *Pandemic*

## Abstract

**Objectives:**

The coronavirus-disease-2019 (COVID-19) pandemic has led to the restructuring of health-services to prioritize the treatment of COVID-19. The severe restrictions on daily life affected the management of chronic diseases. Patients with a previous history of premature myocardial infarction (MI) are a vulnerable group requiring frequent and continued medical attention both in the pandemic and non-pandemic era. The present study was conducted to provide insight into the impact of COVID-19 outbreak on heart-healthy lifestyle and management of patients with a history of premature MI.

**Methods:**

This cross-sectional study included 170 consecutive patients with a history of premature MI who were already in regular follow-up in a tertiary out-patient prevention clinic before the pandemic. Inclusion criteria included age ≥18 years and being on regular follow up with the diagnosis of premature MI (documented MI before the age of 55 years) at least for one year. All patients were contacted by phone-call and replied to a 23-item questionnaire measuring the impact of the pandemic on the management, healthy lifestyle habits, and anxiety level.

**Results:**

One patient died due to COVID-19 infection; therefore the analyses were conducted in 169 patients (age: 47.67 ​± ​11.84 years, 21.3% women). The median age at first MI was 39 (IQR 10) years and the median time elapsed since the first MI was 7 years (IQR 10). The study population was highly compliant with the follow-up visits (78.1%) and pharmacological therapy (97%) before the pandemic according to the medical files. The majority (82.2%) of the patients were aware that having a history of premature MI would increase the risk and harm of COVID-19. Anxiety level increased in 62.7% of the study patients. Overall, 65.7% of the patient group reported a disruption at least in ≥1 component(s) of healthy life-behaviors (non-compliance with the heart-healthy diet, an increase in alcohol intake, an increase in smoking, and/or reduced physical activity) since the emergence of the outbreak. The anxiety level (p ​= ​0.001) and the prevalence of appetite change (p ​< ​0.0001) and weight gain (p ​< ​0.0001) was lower in the lifestyle compliant group than the non-compliant group. Avoidance of seeking medical care was reported in 33.7% of the patients. Statin use was 99.4% before the pandemic and decreased to 89.9% (p ​< ​0.0001) ​despite the fact that medications were reimbursed and widely available.

**Conclusions:**

The COVID-19 pandemic significantly affected the heart-healthy lifestyle and anxiety levels of patients with a history of premature MI who were already in regular follow-up in a tertiary prevention clinic and led to significant avoidance of medical care. More rigorous follow-up, education, and reassurance of these patients with telemedicine are necessary for the prevention of further increase in their risk.

## Graphical abstract

**Figure:**
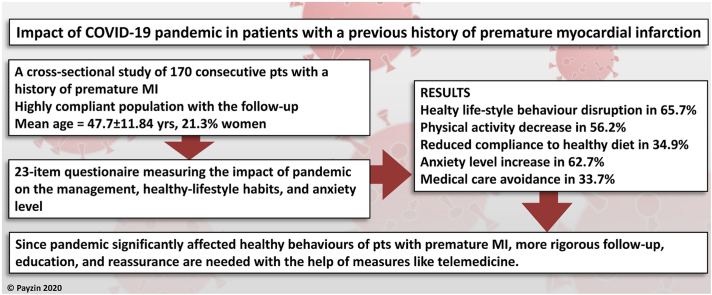

## 1 Introduction

The sudden outbreak of Coronavirus Disease-2019 (COVID-19), led to severe restrictions on daily life including lockdown and community containment measures affecting the management of patients with chronic diseases. Patients with chronic disease including cardiovascular (CV) disease were documented to be more prone to adverse outcomes of COVID-19 with significantly increased mortality even at early ages [1,2]. With an increased risk of inflammation, thrombosis, and atherosclerosis, patients with a previous history of premature myocardial infarction (MI) are a vulnerable group requiring frequent and continued medical attention both in the pandemic and non-pandemic era. To effectively treat CV disease, besides compliance with pharmacologic therapy, heart-healthy lifestyle measures have to be prioritized in these patients [4]. The present study was conducted to investigate the impact of the COVID-19 outbreak on adherence to the healthy lifestyle, management, and anxiety levels of patients with a history of premature MI.

## 2 Materials and methods

This cross-sectional study included 170 consecutive patients with a history of premature MI currently followed at the ‘Young MI Outpatient Clinic’ of Ege University Medical School Cardiology Department. Patients with premature MI are followed up in a separate secondary prevention clinic in our department since they constitute a special group of patients with high risk for future events and frequently have genetic dyslipidemias. The study protocol was approved by both the Institutional Review Board (May 15, 2020, 20-5 ​T 49) and the Ministry of Health COVID-19 Scientific Research Oversight Committee. Informed consent was obtained from all participants.

Inclusion criteria included age ≥18 years and being in regular follow up with the diagnosis of premature MI at least for one year. Premature MI was defined as having documented MI before the age of 55 years. Cognitive dysfunction preventing informed consent was accepted as an exclusion criterion. Patients not accepting the use of their data for the study, and patients who could not be contacted with at least three phone calls were also excluded.

Data were collected by phone contacts from the participants between 15–25 May 2020. All patients were requested to reply to a survey consisting of 23 questions (Supplementary Table 1). The questionnaire was constructed by a cardiologist (MK) and a psychiatrist (OKT) who are both experienced in survey preparation. This interview-based questionnaire targeted to understand the impact of COVID-19 pandemic on management, healthy lifestyle habits (sleeping, exercise, smoking, alcohol consumption, healthy diet, etc.), anxiety levels, and adherence to personal protection measures (PPM) of patients with a history of premature MI. The survey also explored if there was any disruption in the treatment due to the COVID-19 epidemic. Additionally, patients were questioned whether they would go to the hospital if they had a severe complaint such as chest pain during the pandemic period. Surveys were conducted by a trained experienced research secretary. Clinical CV characteristics of the patient group were obtained from the medical files of the ‘Young MI Outpatient Clinic’. The retrieved information included demographic characteristics, medical history, age at the first acute coronary event, history of coronary revascularization, ejection fraction (EF), last visit date, CV risk factors, family history of coronary artery disease (CAD), physical examination findings, compliance with treatment, and pretreatment and the latest low-density lipoprotein (LDL)-cholesterol levels, and HbA_1c_ levels.

Diabetes mellitus was defined as a fasting plasma glucose level of ≥126 ​mg/dL, or a history of diagnosis/treatment for diabetes, or glycated hemoglobin (HbA_1c_) ≥6.5% if available. Hypertension was defined as the presence of resting systolic blood pressure ≥140 ​mmHg, or diastolic blood pressure ≥90 ​mmHg on at least 2 occasions, or a history of diagnosis/treatment of hypertension. Hypercholesterolemia was defined as fasting total cholesterol ≥200 ​mg/dL or LDL-cholesterol ≥130 ​mg/dL or treatment with any cholesterol-lowering drug, without other underlying conditions that increase cholesterol, such as hypothyroidism or pregnancy. Patients with a history of CAD in any first or second-degree relatives under the age of 55 years for males and 65 years for females were accepted as positive for a family history of CAD.

All statistical analyses were conducted using SPSS Version 22.0 (SPSS Inc., Chicago, IL). Shapiro-Wilk test was used to test normality. Normally distributed quantitative data were presented by mean and standard deviation, and the data not normally distributed were expressed by the median and minimum-maximum values, or interquartile range (IQR). Categorical variables were presented by frequencies or percentages. Student t-test was used for comparisons of the quantitative data with normal distribution, the Mann Whitney *U* test, and the Kruskal Wallis test were used for comparing the data not distributed normally. Dunn test with Bonferroni adjustment was applied for nonparametric post hoc procedure. For comparison of the qualitative data, the *X*^*2*^ test or Fisher’s-exact tests were used. Pearson-correlation analysis was used to assess correlations between continuous variables with normal distribution, and Spearman’s rank test was used for the variables with skewed distribution. A p-value of <0.05 (two-sided) was considered statistically significant.

## 3 Results

### 3.1 Population characteristics

A total of 170 patients with premature MI were contacted by phone for the present cross-sectional study. As 1 patient died due to COVID-19 infection, the analysis was conducted for 169 patients (mean age: 47.67 ​± ​11.84 years, 21.3% women) who replied to all the questions. Table 1 displays the clinical characteristics and questionnaire details of the study population. The last cardiology outpatient visit before the pandemic period was 5 (IQR 4.5) months ago and 21.89% of the patients had control visits within the last 3 months before the emergence of the pandemic. The majority of the study population were highly compliant with the follow-up visits (78.1%) and pharmacological therapy (97%) according to the medical files before the pandemic. Statin use was 99.4% before the pandemic and decreased to 89.9% (p ​< ​0.0001) despite the fact that medications were reimbursed and available. Of the whole study population, only 2 patients acquired COVID-19, and one recovered with renal failure requiring hemodialysis and one without any sequelae.

**Table 1:** **Clinical characteristics and survey results of the study population**.

N	169
Mean age, years	47.67 ​± ​11.84
Female, n (%)	36 (21.3)
University and/or higher education, n (%)	90 (53.3)
Median age at the first MI, years	39 (IQR:10)
Median time elapsed since the first MI, years	7 years (IQR:10)
Median time elapsed since the last cardiology outpatient visit before the pandemic period, months	5 (IQR:4.5)
Median time elapsed since the last coronary angiography, years	4 (IQR:5)
Median LDL-cholesterol levels before the pandemic (mg/dL)	
Pre-treatment (n ​= ​163)	153 (IQR:96)
Last measurement (n ​= ​159)	88 (IQR:59)
Median HbA1c levels in the last year (%) (n ​= ​46)	6.1 (IQR:1.4)
Median ejection fraction (%)	55 (IQR:15)
Compliance with pharmacologic therapy before the pandemic, n (%)	164 (97)
Compliance with follow-up visits before the pandemic, n (%)	132 (78.1)
Statin use during the pandemic,	152 (89.9)
History of revascularization, n (%)	
Coronary bypass grafting	44 (26)
Percutaneous coronary intervention	118 (69.8)
Cardiovascular risk factors, n (%)	
Hypertension	68 (40.2)
Hypercholesterolemia	121 (71.6)
Obesity	51 (30.2)
Current smoking	122 (72.2)
Diabetes mellitus	43 (25.4)
Family history of CAD	139 (82.2)
Any current complaint during the pandemic	14 (8.3)
**Questionnaire items**	
Working status, n (%)	
Unemployed	71 (42.0)
Do not work due to the pandemic	61 (36.1)
Work from home	9 (5.3)
Going to workplace	28 (16.6)
Anxiety level (Median-IQR)	5–5
Increase in anxiety level, n (%)	106 (62.7)
% for compliance to the recommended personal protective measures (Median-IQR)	90–20
Sleep duration, n (%)	
Increased	27 (16.0)
Decreased	32 (18.9)
No-change	110 (65.1)
Appetite, n (%)	
Changed (decreased or increased)	59 (34.9)
No change	110 (65.1)
Weight gain, n (%)	51 (30.2)
Compliance with Diet, n (%)	
Worse	59 (34.9)
Better	35 (20.7)
No change	75 (44.4)
Exercise habits, n (%)	
Worse	95 (56.2)
Better	31 (18.3)
No change	43 (25.4)
Smoking, n (%)	
Non-user	122 (72.2)
Smoker	47 (27.8)
Increased	12 (7.1)
Decreased	21 (12.4)
No change	14 (8.3)
Alcohol consumption, n (%)	
Non-user	144 (85.2)
Consumer	25 (14.8)
Increased	7 (4.1)
Decreased	6 (3.6)
No change	12 (7.1)
Being admitted to a hospital during pandemic, n (%)	25 (14.8)
Would admit to a hospital in case of a complaint, n (%)	112 (66.3)

CAD: coronary artery disease, SD: standard deviation, IQR: interquartile range, LDL: Low density lipoprotein.

### 3.2 Effect of the pandemic on heart-healthy lifestyle

Overall, 65.7% of the patients reported a disruption at least in one or more components of the healthy life behaviors (non-compliance with the heart-healthy diet, increase in alcohol intake, increase in smoking, and/or reduced physical activity). Those patients who were non-compliant to the healthy life-style during the pandemic reported more complaints in the outbreak period and higher anxiety levels. Also, the prevalence of decreased sleep was higher in non-compliant patients whereas no sleep change was higher in lifestyle compliants (Table 2). Compliance with a healthy diet was decreased in more than a third of the patients (34.9%) meanwhile only 20.7% were trying to be more compliant with diet since the emergence of the outbreak (Table 1). More than half of the patients (56.2%) reported a reduction in physical activity.

**Table 2:** Comparison of the patients who are compliant to healthy life style and the non-compliant group during the pandemic period.

Variables	Life-style compliant (n ​= ​58)	Life-style non-compliant∗ (n ​= ​111)	Statistics
Median age, (IQR)	48.5 (14.3)	46 (18)	p ​= ​0.210
Female, n (%)	15 (25.9)	21 (18.9)	p ​= ​0.396
University or higher education, n (%)	30 (51.7)	60 (54.1)	p ​= ​0.773
Working status			NA
a. Unemployed	27 (46.6)	44 (39.6)	
b. Do not work due to the pandemic	21 (36.2)	40 (36)	
c. Work from home	1 (1.7)	8 (7.2)	
d. Going to workplace	9 (15.5)	19 (17.1)	
Median age at the first MI, years, (IQR)	40 (9.25)	39 (9)	p ​= ​0.162
Median time elapsed since the first MI, years, (IQR)	8 (11)	7 (9)	p ​= ​0.983
Median time elapsed since the last cardiology outpatient visit before the pandemic period, months, (IQR)	4.1 (5.1)	4 (5)	p ​= ​0.855
Median time elapsed since the last coronary angiography, years, (IQR)	4.6 (3.5)	5 (8)	p ​= ​0.991
Median LDL-cholesterol levels before pandemic (mg/dL), (IQR)			
Pre-treatment	148.5 (128.25)	153 (88)	p ​= ​0.877
Last measurement	81 (52)	91 (69.25)	p ​= ​0.314
Median HbA1c levels in the last year (%), (IQR) (n ​= ​46)	5.75 (0.8)	5.8 (1)	p ​= ​0.331
Median ejection fraction (%), (IQR)	55 (15)	60 (15)	p ​= ​0.431
History of CABG, n (%)	17 (29.3)	27 (24.3)	p ​= ​0.605
History of PCI, n (%)	37 (63.8)	81 (73)	p ​= ​0.290
Hypertension, n (%)	25 (43.1)	43 (38.7)	p ​= ​0.701
Hypercholesterolemia, n (%)	40 (69)	81 (73)	p ​= ​0.712
Obesity, n (%)	16 (27.6)	35 (31.5)	p ​= ​0.723
Diabetes mellitus, n (%)	14 (24.1)	29 (26.1)	p ​= ​0.924
Family history of CAD, n (%)	48 (82.8)	91 (82)	p ​= ​1
Statin use during the pandemic, n (%)	53 (91.4)	99 (89.2)	p ​= ​0.857
Compliant with pharmacologic therapy, n (%)	56 (96.6)	108 (97.3)	p ​= ​1
Compliant with follow-up visits, n (%)	47 (81)	85 (76.6)	p ​= ​0.639
Any current complaint n (%)	1 (1.7)	13 (11.7)	**p ​= ​0.036**
Median anxiety level (IQR)	3 (4)	6 (6)	**p ​= ​0.001**
% for compliance to the recommended personal protective measures, Median (IQR)	90 (20)	90 (20)	p ​= ​0.727
Increased anxiety, n (%)	30 (51.7)	76 (68.5)	**p ​= ​0.049**
Thought of increased vulnerability within the course of pandemic due to suffering MI at young age, n (%)			
a) Yes	47 (81)	92 (82.9)	p ​= ​0.653
b) No	7 (12.1)	9 (8.1)	
c) Do not know	4 (9%)	10 (6.9)	
Sleep duration, n (%)			**p ​= ​0.010**
a) Increased	7 (12.1)	20 (18)	a-b: p ​= ​0.513
b) Decreased	5 (8.6)	27 (24.3)	a-c**:** p ​= ​0.194
c) No-change	46 (79.3)	64 (57.7)	**b-c: p ​= ​0.012**
Appetite changed, n (%)	5 (8.6)	54 (48.6)	**p ​< ​0.0001**
Weight gain, n (%)	4 (6.9)	47 (42.3)	**p ​< ​0.0001**
Being admitted to a hospital during pandemic, n (%)	4 (6.9)	21 (18.9)	p ​= ​0.063
Would admit to a hospital in case of a complaint, n (%)	36 (62.1)	76 (68.5)	p ​= ​0.507

CAD: Coronary artery disease, IQR: interquartile range, NA: non-applicable, LDL: low density lipoprotein, MI: myocardial infarction, CABG: Coronary bypass grafting, PCI: Percutaneous coronary intervention.

∗Non-compliant defined as a disruption at least in one or more components of the healthy life behaviors (non-compliance with the heart healthy diet, increase in alcohol intake, increase in smoking, and/or reduced physical activity).

The majority of the patients were already non-smokers and were not in the habit of taking alcohol in any form. With the introduction of COVID-19 breakout alcohol consumption was increased in only 7 patients. The pandemic also affected the sleeping habit, almost one-third of the patients reported a change in sleep duration.

### 3.3 Anxiety and adherence to personal protection measures during the pandemic

Almost two-thirds of the study population reported an increase in anxiety with the COVID-19 pandemic. The factors which are related to the anxiety level are presented in Table 3. Patients who were aware of the increased risk of COVID-19 due to their history of MI at a young age had higher anxiety than the remaining patients (p ​< ​0.0001). The increase in anxiety after the emergence of the pandemic was associated with a change in diet (p ​= ​0.024) and statin use (p ​= ​0.001).

**Table 3:** Comparison of patient reported anxiety levels according to analyzed factors during the pandemics.

		Anxiety level		Statistics
		Median	IQR	
Gender	Female	5	5	p ​= ​0.739
	Male	5	5	
Education	Lower than university	5	5	p ​= ​0.776
	University and/or higher	5	5	
Working status	Unemployed	5	5	p ​= ​0.264
	Do not work due to the pandemic	3	5	
	Work from home	6	7	
	Going to workplace	5	3,8	
History of CABG	+	5.5	4	p ​= ​0.236
	-	5	5	
History of PCI	+	5	5	p ​= ​0.254
	-	4	5	
Diabetes mellitus	+	5	5	p ​= ​0.256
	-	5	5	
Hypertension	+	5	5	p ​= ​0.927
	-	5	5	
Hypercholesterolemia	+	5	4	**p ​= ​0.026**
	-	4	4	
Obesity	+	5	4	p ​= ​0.737
	-	5	5	
Family history of CAD	+	5	4	**p ​= ​0.001**
	-	3	4	
Complaint during pandemic	+	7.5	5	**p ​= ​0.003**
	-	5	5	
Drug use properly during the outbreak	Yes	5	5	p ​= ​0.059
	No	3	3.8	
Contracting SARS-CoV-2	Yes	7		p ​= ​0.400
	Do not know	2		
	No	5	5	
COVID-19 diagnosis in the family	Yes	8		p ​= ​0.311
	No	5	5	
Compliant to follow-up visits	Yes	5	5	p ​= ​0.722
	No	5	4.5	
Thought of increased vulnerability within the course of pandemic due to suffering MI at a young age	a.Yes	6	4	**p ​< ​0.0001**
	b.Do not know	2	2.3	**a-b: p ​< ​0.0001**
	c.No	2	1	**a-c: p ​< ​0.0001b-c: p ​= ​1**
Sleep duration	a.Increase	5	4	**p ​= ​0.002**
	b.Decrease	7	4.8	a-b: p ​= ​0.222
	c.No change	4	5	a-c: p ​= ​0.855**b-c: p ​= ​0.002**
Appetite	a.Eating more	5	6	**p ​= ​0.001**
	b.Eating less	8	3	**a-b: p ​= ​0.018**
	c.No change	4.5	4	a-c: p ​= ​0.430**b-c: p ​= ​0.001**
Weight gain	Yes	4	6	p ​= ​0.977
	No	5	5	
Diet	Worse	6	6	p ​= ​0.337
	Better	5	6	
	No change	5	4	
Exercise habits	a.Worse	6	5	**p ​= ​0.005**
	b.Better	3	4	**a-b: p ​= ​0.012**
	c.No change	3	5	a-c: p ​= ​0.070b-c: p ​= ​1
Smoking amount	Non-user	5	5	p ​= ​0.079
	Increased	7.5	3.8	
	Decreased	4	3	
	No change	2.5	5.3	
Alcohol consumption	Non-user	5	5	p ​= ​0.555
	Increased	7	4	
	Decreased	3.5	6	
	No change	5.5	5	
Being admitted to a hospital during pandemic	Yes	6	5	p ​= ​0.100
	No	5	5	
Would admit to a hospital in case of a complaint	Yes	5	5	p ​= ​0.155
	No	5	5	

CAD: Coronary artery disease, IQR: interquartile range, MI: myocardial infarction, CABG: Coronary bypass grafting, PCI: Percutaneous coronary intervention.

The median level of compliance with the recommended PPM were 90%. The age, the duration since the first MI, duration since the last control visit, the duration since the last coronary angiography, and the HbA1C levels were not correlated with both the levels of anxiety and the compliance to PPM (Supplementary Table 2). EF was negatively correlated with the level of compliance to PPM (p ​= ​0.049, r ​= ​−0.152), but not correlated with the patient-reported anxiety level (p ​= ​0.341, r ​= ​−0.074).

Compliance with PPM was not associated with any characteristics or reported changes except for the working status of the patient group (Supplementary Table 2).

### ***3*.4** **Medical care avoidance during the pandemic and awareness of the individual risk of COVID-19.**

Most of the patients (82.2%) were aware that having a history of premature MI would increase the risk and/or harm of COVID-19. During the COVID-19 outbreak, 25 (14.8%) patients sought medical care with cardiac (56%) and extra-cardiac (44%) symptoms. One-third of the patients (33.7%) stated that they would not seek medical care if they had a complaint such as chest pain during the COVID-19 outbreak. In all those with avoidance of seeking medical care, the reason was the fear of contracting coronavirus at the healthcare services. Interestingly, medical care avoidance was significantly more prominent in women (p ​= ​0.033).

Awareness about the vulnerability of COVID-19 was associated with “increased anxiety” (p ​< ​0.0001). The proportion of patients who reported increased anxiety was 94.3% in the patient population with better awareness, 2.8% in the patient group claiming that they had no increased risk and again 2.8% in the patient group who have no idea. The prevalence of statin use (92.8% vs 76.7%, p ​= ​0.015) and presence of a family history of CAD (85.6% vs 66.7%, p ​= ​0.028) were higher in the group of patients with better awareness. Also, patients with better awareness were more compliant with their pharmacotherapy than the remaining patients (97.1% vs 86.7%; p ​= ​0.034).

### **3.5** **The effect of being compliant with regular follow-up before the pandemic period.**

We also explored if being compliant with follow-up visits and/or adherence to the use of CV medications before the pandemic period had any effect on the changes reported on healthy lifestyle and anxiety levels during the COVID-19 days. As expected, females were more compliant (91.7% vs 74.4%, p ​= ​0.047). Smoking (80% vs 26.2%, p ​= ​0.022), obesity (45.9% vs 25.8%; p ​= ​0.031), and improper drug use during the outbreak (13.5% vs 2.3%, p ​= ​0.013) were more prevalent in the previously less compliant group whereas better diet compliance during the outbreak was more prevalent in the compliant group (24.2% vs 8.1%; p ​= ​0.031). The proportion of patients on statin therapy was also lower in the non-compliant group during the breakout. The non-compliant group had lower anxiety levels (Table 3).

## 4 Discussion

The results of this descriptive survey showed that the COVID-19 pandemic significantly affected the heart-healthy lifestyle and anxiety levels of patients with a history of premature MI who were already in regular follow-up in a tertiary out-patient prevention clinic before the pandemic. None of the study population had problems in access to treatment. With the initiation of the pandemic, while quarantine measures and restrictions were introduced to avoid an unnecessary visit to hospitals and to prevent disruption of chronic treatments the Ministry of Health, enabled patients to access drugs for chronic diseases from pharmacies without new prescriptions for 3 months [5]. This action was a very effective precaution to avoid interruption of pharmacotherapy in the management of chronic diseases. Despite this precaution, statin use decreased in such a previously compliant patient group.

During the pandemic, 65.7% of the study population reported decreased compliance to at least one or more components of healthy life behaviors including non-compliance with the heart-healthy diet, increase in alcohol intake, increase in smoking, and/or reduced physical activity. The current COVID-19 crisis with social distancing, lockdown, and telecommuting reduced exercise and mobility options, markedly decreased levels of physical activity [4]. Staying at home with a sedentary lifestyle and enormously increased screen-time decreased energy consumption meanwhile increasing the difficulty to access fresh fruit and vegetables hampering most heart-healthy eating habits. More than half of the patients reported reduced physical activity, meanwhile, only 18.3% reported an increased level of exercise. In particular, almost 1/3rd of our patients reported increased appetite, 30.2% weight gain, and 34.9% decreased compliance to a healthy diet during the outbreak. Only one-fifth of the patients were trying to be more compliant with diet during the pandemic days. As the study population represents a high treatment adherent group of patients, the observed negative impact on the healthy lifestyle possibly would be deeper in the general CAD population. Several weeks of a reduction in physical activity and daily step-count combined with modified eating habits (physical inactivity plus overfeeding) have metabolic consequences such as increases in insulin resistance, total body fat, abdominal fat, and inflammatory cytokines [6].

The COVID-19 outbreak caused a significant increase in anxiety level as reported in almost two-thirds of our study patients. Interestingly, age, the duration since the first MI, duration since the last control visit, and the duration since the last coronary angiography were not correlated with the anxiety level. It is noteworthy that patients with higher anxiety levels were sleeping less, eating less, and having less physical activity. On the other hand, we found that patients who do not perceive themselves as vulnerable had lower anxiety levels than the ones who were aware that CAD patients were at higher risk for COVID-19 complications. This, relatively low anxiety level, seems to be a result of the lack of knowledge leading to lower risk perception. Consistent with our findings Lee et al. found that participants with optimistically biased perception experienced lower anxiety than the remaining participants, while all the participants were at risk of CV disease [7]. Perceptions of threat and availability of coping sources are predictors of health-related behaviors [8]. Likewise, patients perceiving themselves vulnerable were more compliant to their pharmacotherapy. It seems that optimum anxiety level yields effective coping strategies and preventive measures such as having higher physical activity. On the other hand, we have to mention that severe anxiety levels might yield to poor prognosis as it was revealed that patients with generalized anxiety disorder have increased major adverse cardiac event risk [9]. A recent study including patients attending a cardiac rehabilitation program concluded that greater baseline levels of traumatic stress related to poor cardiac outcomes such as higher fasting blood glucose and HbA1C [10].

A noticeable finding of our study is that 33.7% of the study population reported that they would not seek medical care if they had a complaint such as chest pain during the COVID-19 outbreak due to the fear of contracting coronavirus. This observation is extremely important as it provides real-life evidence and denotes the underlying cause of the late and reduced number of admissions of patients presenting with acute coronary syndromes reported during the COVID-19 breakout [11,12]. The picture should be more pronounced for the general CAD and/or high CV risk populations as our study population constituted of patients with very high health literacy and highly aware patients of their own CV risk in regular follow-up in a tertiary prevention clinic. Medical care avoidance was significantly more prominent in women. This finding is in line with the previous observation during the H1N1 outbreak that females have higher emotional distress and avoidance of hospitals [13]. Therefore, women should be paid more attention during the current and possible following pandemics.

The overall statin use declined to 89.9% in the patient population which was very compliant (99.4%) before the pandemic. Adherence to statin treatment was associated with the family history of CAD, adherence to follow-up visits and treatment before the pandemic period, anxiety level, and the awareness of the increased risk of COVID-19 due to early CAD in the study population.

All our findings indicate the importance of being in contact with the patients. The previous outbreaks have also clearly demonstrated that optimum anxiety and high perception of risk were associated with taking preventive measures whereas risk uncertainty and disbelief in media reports were associated with low engagement in the recommendations [8,14,15]. Therefore, preventive cardiology programs with telemedicine should be in place for remote monitoring of the high CV risk patients to optimize risk factor control, physical activity levels, and assess diet while isolated [3,16]. Such programs besides providing the necessary information also increase the adherence to CV medications. Both the national scientific societies and patient organizations may also support patients by providing reliable, accurate information and advice in this time of greatest need [3,16]. Social media is also crucial in increasing public awareness for overcoming the patients’ fear of contracting the coronavirus leading to medical care avoidance. For example, the recent ‘When your heart says so #JustGo’ campaign organized by the patient advocacy groups (FH Europe, Global Heart Hub) and supported by the European Atherosclerosis Society reached by ​> ​80,000,000 people on social media and encouraged patients to seek medical help in case of cardiac symptoms [16].

This study has several limitations. The low number of patients could be accepted as a limitation but conducting a phone call based survey of 170 patients during the pandemic days was challenging. Moreover, the study population consisted of highly compliant patients with premature MI receiving long-term care in a special prevention clinic from which the results might not be subject to generalization. And our results are probably underestimating the attitude/behavioral changes in the general CAD population during the pandemic period. Moreover, as our center is also an experienced heart transplantation center, patients with functional class 3 or more, are followed in the heart failure and transplantation unit. Therefore, though low EFs all the patients in the young MI clinic are stable with regard to functional class without overt heart failure. It’s expected that EF may influence the response of the patient population to the pandemic. We only found a weak negative correlation with limited significance between EF and compliance to PPM (p ​= ​0.049, r ​= ​−0.152). The constitution of the study population of a heterogeneous group with good functional class could be the explanation for not finding any association with EF and the studied measures during the pandemic. Lack of a control group might also be accepted as a limitation. To assess the impact of the COVID-19 pandemic in patients with a previous history of premature MI, a matched cohort of patients without a history of premature MI would be the real control [17]. However, during the COVID-19 era, such a control group consisting of >55 years of age would have a higher risk for the infection, and would probably be more careful about the lifestyle and PPM. Therefore, we focused on the specific findings unique to those with a history of premature MI. Also, this study methodologically could harbor some degree of bias like all surveys. To overcome the patients’ possibility of replying in favor to please their physicians, a trained secretary experienced in surveys performed the questionnaire. As there were no validated COVID-19 questionnaires with regard to life-style and anxiety at the time of the conduction of the study, the questions were generated by an experienced team consisting of a cardiologist (MK) and a psychiatrist (OKT). And finally, the results could vary during the time course of the pandemic. However, we especially called patients at least 1 month after the diagnosis of 1st patient with COVID-19 in the country, to ensure the adaptation of the patients to the preventive measures and quarantine precautions.

Our acceptance of the definition of premature MI as age <55 years, could be a matter of debate. As there is no consensus for a universally accepted age cut-off for defining young MI, there is a disparity in the literature; some studies vary from ≤40 to ≤55 years of age and others define young MI as <45 years of age [[18], [19], [20], [21], [22]]. We accepted the Framingham cohort’s cut off which has defined premature MI as < 55 years of age which is also the adopted definition of premature MI for men and women by CV disease management algorithms established by the Ministry of Health in our country [21].

In conclusion, the COVID-19 pandemic significantly affected the heart-healthy lifestyle and anxiety levels of patients with a history of premature CAD who is already in regular follow-up in a tertiary prevention clinic and caused a significant avoidance in seeking medical care. Therefore, public education and better measures such as telemedicine, and COVID-free centres for patients with chronic diseases are necessary to afford a regular follow-up, control of adherence to therapy, and to ensure not to increase further risk, especially in the case of prolongation of the COVID-19 pandemic.

## COI statement

- Meral Kayikcioglu has received honoraria (for lectures and consultancy) from Abbott, Actelion, Astra-Zeneca, Abdi Ibrahim, Aegerion, Bayer Schering, Menarini, Sanofi Genzyme and Pfizer, and research funding from Aegerion, Amyrt Pharma, Amgen, Pfizer, and Sanofi and has participated in clinical trials with Amgen, Bayer Schering, Sanofi, and for the last 3 years.
- Ozlem Kuman Tuncel has no conflicts of interest.
- Lale Tokgozoglu has received honoraria/consultancy fees from Merck, Amgen, Astra, Novartis, Abbott, Daichi Sankyo, Nova Nordisk, Pfizer, Actelion, Servier, Sanofi, Boehringer Ingelheim, Menarini, Kowa, Aegerion, and Abbott.
