# Supplementary material for: Impact of the COVID-19 pandemic in patients with a previous history of premature myocardial infarction
Source: Am J Prev Cardiol. 2020 Nov 18;4:100128. doi: 10.1016/j.ajpc.2020.100128 (PMC7673226; doi:10.1016/j.ajpc.2020.100128)
Supplement: Supplementary file 1 [file mmc1.docx]

**Supp table 1. Study Questionnaire**

|  |  | **Multiple choices** |
| --- | --- | --- |
| **Q1** | **Do you have any complaint during the epidemic?** | **Yes/No** |
| **Q2** | **Could you use your medicines regularly during the epidemic?** | **Yes/No/ Sometimes disrupted** |
| **Q2b** | **If Q2 is yes then Why can't you take your medication?** |  |
| **Q3** | **When was your last admission to a CV prevention clinic?** | **-** |
| **Q6** | **Are you working during the epidemic?** | 1. **not working before the pandemic** 2. **not working because of the pandemic** 3. **working from home** 4. **working** |
| **Q7** | **Have you had COVID-19 disease?** | 1. **Yes - I got the diagnosis, I received treatment** 2. **No** 3. **Do not know** 4. **I had flu complaints, but I did not go to the doctor,** |
| **Q8** | **Did any of your family members have COVID infection?** | **Yes/No/don’t know** |
| **Q9** | **Do you think that COVID-19 may affect you worse or cause more harm because you have premature myocardial infarction in the past?** | **Yes/No/don’t know** |
| **Q10** | **Did your anxiety increased during the COVID outbreak period?** | **Yes/No** |
| **Q11** | **What is your anxiety level due to COVID outbreak? Please define it as a score ranging from 1 to 10. (1 represents the lowest and 10 represents the highest scores)** | **-** |
| **Q12** | **Did you experience any change in your sleep since the emergence of the outbreak?** | **a. Yes, I sleep less**  **b. Yes, I sleep more**  **c. No** |
| **Q13** | **Has there been any change in your appetite since the emergence of the outbreak?** | **Yes/No** |
| **Q14** | **Has your compliance with your diet changed since the emergence of the outbreak?** | **a. Yes, I pay more attention**  **b. Yes, I can't follow my diet**  **c. No** |
| **Q15** | **Has there been any change in your exercise habits since the emergence of the outbreak?** | **a. Yes, I exercise more**  **b. Yes, I exercise less**  **c. unchanged** |
| **Q16** | **If you are smoking, has there been a change in your amount of smoking since the emergence of the outbreak?** | **a. No, I am not a smoker**  **b. Yes, I smoke more**  **c. Yes, I smoke less**  **D. Unchanged** |
| **Q18** | **If you drink alcohol, has there been a change in the amount you drink since the emergence of the outbreak?** | **a. No, I am not a smoker**  **b. Yes, I smoke more**  **c. Yes, I smoke less**  **D. Unchanged** |
| **Q19** | **Up to what percent you can comply with the** **personal protection measures (using mask, social distancing of 1 m, frequent hand washing etc.)?** |  |
| **Q21** | **Did you admit to a hospital or a health center with any complaint during the epidemic?** | **Yes/No** |
| **Q22** | **If you had a complaint (for example chest pain) would admit to a hospital during the COVID outbreak?** | **Yes/No** |
| **Q23** | **Do you have any additional problem that you want to mention?** | **-** |
