# Supplementary material for: Impact of the COVID-19 pandemic in patients with a previous history of premature myocardial infarction
Source: Am J Prev Cardiol. 2020 Nov 18;4:100128. doi: 10.1016/j.ajpc.2020.100128 (PMC7673226; doi:10.1016/j.ajpc.2020.100128)
Supplement: Supplementary file 2 [file mmc2.docx]

| Supplementary Table 2: Comparison of the percentage of adherence to recommended personal protective measures according to analyzed factors | | | | |
| --- | --- | --- | --- | --- |
|  |  | % of adherence to protective measures | | Statistics |
|  |  | Median | IQR |  |
| Gender | Female | 90 | 20 | p= 0.859 |
|  | Male | 90 | 20 |  |
| Education | Lower than university | 90 | 20 | p= 0.558 |
|  | University and/or higher | 90 | 20 |  |
| Working status | a. Unemployed | 90 | 15 | **p=0.004**  a-b: p= 0.886  a-c: p= 0.002  a-d: p=1  b-c: p= 0.002  b-d:1  c-d:0.010 |
|  | b.Do not work due to the pandemic | 90 | 22,5 |  |
|  | c.Work from home | 100 | 0 |  |
|  | d.Going to workplace | 82.5 | 20 |  |
| History of CABG | + | 97 | 20 | p= 0.086 |
|  | - | 90 | 20 |  |
| History of PCI | + | 90 | 20 | p= 0.328 |
|  | - | 90 | 20 |  |
| Diabetes mellitus | + | 90 | 20 | p= 0.727 |
|  | - | 90 | 20 |  |
| Hypertension | + | 90 | 20 | p= 0.691 |
|  | - | 90 | 20 |  |
| Hypercholesterolemia | + | 90 | 20 | p= 0.381 |
|  | - | 90 | 20 |  |
| Obesity | + | 90 | 20 | p= 0.968 |
|  | - | 90 | 20 |  |
| Family history of CAD | + | 90 | 20 | p= 0.582 |
|  | - | 90 | 20 |  |
| Complaint | + | 90 | 20 | p= 0.474 |
|  | - | 90 | 20 |  |
| Drug use properly | Yes | 90 | 20 | p= 0.347 |
|  | No | 80 | 21.8 |  |
| Contracting SARS-CoV-2 | Yes | 60 |  | p= 0.228 |
|  | Do not know | 90 |  |  |
|  | No | 90 | 20 |  |
| COVID-19 diagnosis in the family | Yes | 80 |  | p= 0.686 |
|  | No | 90 | 20 |  |
| Compliant to follow-up visits before pandemic period | Yes | 90 | 20 | p= 0.905 |
|  | No | 90 | 20 |  |
| Thought of increased vulnerability within the course of pandemic due to suffering MI at a young age | Yes | 90 | 20 | p= 0.450 |
|  | Do not know | 80 | 26.3 |  |
|  | No | 85 | 28.8 |  |
| Increase in anxiety level | Yes | 90 | 20 | p= 0.595 |
|  | No | 90 | 20 |  |
| Sleep duration | Increase | 85 | 15 | p= 0.249 |
|  | Decrease | 90 | 20 |  |
|  | No change | 90 | 20 |  |
| Appetite | Eating more | 90 | 23.8 | p= 0.875 |
|  | Eating less | 85 | 20 |  |
|  | No change | 90 | 20 |  |
| Weight gain | Yes | 90 | 20 | p= 0.697 |
|  | No | 90 | 20 |  |
| Diet | Worse | 85 | 25 | p= 0.338 |
|  | Better | 90 | 30 |  |
|  | No change | 90 | 20 |  |
| Exercise habits | Worse | 90 | 20 | p= 0.353 |
|  | Better | 90 | 20 |  |
|  | No change | 90 | 20 |  |
| Smoking amount | Non-user | 90 | 20 | p= 0.378 |
|  | Increased | 90 | 18.8 |  |
|  | Decreased | 80 | 30 |  |
|  | No change | 85 | 30 |  |
| Alcohol consumption | Non-user | 90 | 20 | p= 0.162 |
|  | Increased | 80 | 35 |  |
|  | Decreased | 85 | 17.5 |  |
|  | No change | 80 | 27.5 |  |
| Being admitted to a hospital during pandemic | Yes | 90 | 20 | p= 0.778 |
|  | No | 90 | 20 |  |
| Would admit to a hospital in case of a complaint | Yes | 90 | 20 | p= 0.619 |
|  | No | 90 | 20 |  |
| CAD: Coronary artery disease, IQR: interquartile range, MI: myocardial infarction, CABG: Coronary bypass grafting, PCI: Percutaneous coronary intervention | | | | |
